# Ambient climatic conditions and semen quality: a comparative analysis across the periods before, during, and after the COVID-19 pandemic

**DOI:** 10.3389/fendo.2025.1660662

**Published:** 2025-09-16

**Authors:** Heeyon Kim, Soyeong Park, Yebon Kim, Yun Soo Chung, Jin Kyung Baek, Jeongmi Yoon, Chungsoon Ryu, Bo Hyon Yun, Young Sik Choi, Daewoo Pak, Seok Kyo Seo, Yohan Ko

**Affiliations:** ^1^ Department of Obstetrics and Gynecology, Severance Hospital, Yonsei University College of Medicine, Seoul, Republic of Korea; ^2^ Institute of Women’s Life Medical Science, Yonsei University College of Medicine, Seoul, Republic of Korea; ^3^ Department of Computer Science, Yonsei University, Wonju, Republic of Korea; ^4^ Division of Data Science, Yonsei University, Wonju, Republic of Korea; ^5^ Division of Software, Yonsei University, Wonju, Republic of Korea

**Keywords:** semen quality, ambient temperature, relative humidity, COVID-19 pandemic, male fertility, big data analysis, meteorological data

## Abstract

**Introduction:**

Ambient temperature and humidity can influence male reproductive function; however, it is unclear whether pandemic-related lifestyle and environmental changes modify this climatic susceptibility.

**Methods:**

A total of 2,672 semen analysis records from 1,287 Korean men collected between 2018 and 2024 were analyzed. Data analyses were conducted on a cohort in which some participants underwent multiple semen analyses across different visits. Semen quality data were linked to regional meteorological records for temperature and humidity across two exposure windows: 0–90 days and 70–90 days prior to semen collection. To address intra-subject correlations from repeated measurements, generalized linear mixed-effects models with a Tweedie distribution and log link function were applied. Associations between lagged environmental exposures and semen parameters—including volume, sperm concentration, motility, strict morphology, and total sperm count (TSC)—were evaluated. Subgroup analyses were further performed for the before COVID-19, during COVID-19, and after COVID-19 periods.

**Results:**

Sperm concentration and TSC significantly increased during the COVID-19 pandemic, whereas semen volume declined over time. Sperm concentration increased after the COVID-19 period, compared to the before COVID-19 era. Elevated ambient temperature, particularly during the 70–90-day spermatogenesis-sensitive window, was significantly associated with decreased sperm concentration and TSC; however, only during the COVID-19 period. No consistent association between humidity and semen parameters was observed.

**Discussion:**

These results suggest that pandemic-related changes may have amplified biological vulnerability to climatic stressors. Overall, semen quality improved during the COVID-19 pandemic; however, our findings indicate that this period was uniquely characterized by increased climatic sensitivity of spermatogenesis. This may reflect altered environmental exposure and lifestyle behaviors, highlighting the complex interactions between public health crises, human behavior, and male reproductive health. Future studies should incorporate detailed indoor climate and occupational exposure data to elucidate these associations further.

## 1 Introduction

Male fertility has become an increasingly important focus of reproductive health, as male-related factors account for nearly 50% of infertility cases, either as a sole cause or in combination with female factors (1). Semen parameters, including sperm concentration, motility, morphology, and volume, are influenced by both intrinsic and extrinsic factors, and semen quality has shown a declining trend in recent decades (2, 3). Intrinsic factors such as congenital and genetic abnormalities, endocrine disorders, advancing age, and systemic illness can directly impair spermatogenesis and semen quality (4). Among extrinsic factors, environmental conditions, including ambient temperature and humidity, play a crucial role in spermatogenesis and semen quality (5–7).

The testes function optimally at temperatures lower than the core body temperature, and elevated scrotal or ambient temperatures may impair sperm production and increase oxidative stress (8, 9). Recent studies have shown that exposure to extremely low or high temperatures can negatively affect semen quality, particularly by decreasing sperm count and motility (10–12). These findings suggest a U-shaped relationship between ambient temperature and male reproductive outcomes, in which the optimal temperature supports better semen quality. Similarly, fluctuations in humidity may influence hormonal balance and thermoregulation, indirectly affecting spermatogenic function (7, 13).

The coronavirus disease 2019 (COVID-19) pandemic has introduced unprecedented shifts in human behavior and societal structures, including psychological stress, altered daily routines, increased sedentary lifestyle, widespread face mask use, and reduced outdoor activity because of social distancing mandates and lockdown measures (14). These societal changes may have had direct and indirect effects on reproductive health (15, 16). Furthermore, stress and systemic illness related to severe acute respiratory syndrome coronavirus 2 infection or COVID-19 messenger ribonucleic acid vaccination have been postulated to disrupt spermatogenesis (17). However, environmental exposures such as temperature and humidity continued to fluctuate regardless of the pandemic phase, creating a unique opportunity to evaluate how climate factors correlate with semen quality across different periods of pandemic.

South Korea, located in a temperate region of East Asia, experiences four distinct seasons—winter (December to February), spring (March to May), summer (June to August), and autumn (September to November) —with substantial intra- and interseasonal fluctuations in temperature and humidity. This climatic diversity provides a naturally varying environmental context that is ideal for investigating how ambient conditions influence human health, including male reproductive function. Such seasonality enables the assessment of environmental stressors across a broad range of temperature and humidity levels, improving our understanding of how extrinsic climatic factors may affect spermatogenesis.

In this retrospective study, we aimed to examine the association between climatic conditions, specifically maximum, average, and minimum temperatures and relative humidity, and semen parameters. We used national meteorological data and semen analysis records collected before, during, and after the COVID-19 pandemic to investigate the temporal and environmental trends in male fertility. Our objective was to assess whether the effects of climate on semen quality differed across the pre-pandemic, pandemic, and post-pandemic periods, thereby offering new insights into the interaction between environmental and societal factors influencing male reproductive health.

## 2 Materials and methods

### 2.1 Study population

This retrospective study was conducted with approval from the Institutional Review Board of Severance Hospital, Yonsei University College of Medicine (Seoul, South Korea, Institutional Review Board #2024-3819-002), and all data were anonymized. Between January 1, 2018, and December 31, 2024, 2,671 semen analysis records were collected from male individuals who attended the Infertility Clinic at the Department of Obstetrics and Gynecology, Severance Hospital, Yonsei University. All available semen analyses conducted during this period were included, regardless of the reason for testing. The study population consisted of men who underwent semen analysis for (1) routine physical examination, (2) assisted reproductive technology procedures, such as intrauterine insemination or *in vitro* fertilization, and (3) sperm banking for fertility preservation. Multiple semen analyses from the same individual were considered as separate records. Repeated tests from the same individuals were analyzed separately to assess within-subject variability concerning meteorological conditions and COVID-19 pandemic phases.

### 2.2 Semen analysis

All participants were instructed to abstain from ejaculation for 2–7 days prior to semen collection. Semen samples were obtained by masturbation into sterile, wide-mouthed containers and processed within 1 h of the collection. To minimize pre-analytical variability because of dehydration, pH instability, and temperature fluctuations, each sample was liquefied at 37°C for 20 min before analysis. Semen analysis was performed using Computer-Assisted Semen Analysis technology (Hamilton Thorne Inc., Beverly, MA, USA) and included the measurement of semen volume, sperm concentration, motility, percentage of morphologically normal sperm, and total sperm count (TSC).

All semen parameters were analyzed according to the World Health Organization laboratory manual for the examination and processing of human semen. Throughout the study period, all analyses were conducted by the same two experienced laboratory technicians using identical protocols, ensuring methodological consistency and minimizing inter-observer variability. Internal quality control and routine external proficiency assessments were performed throughout the study. TSC was calculated as the product of semen volume and sperm concentration (TSC = semen volume [mL] × sperm concentration [×10^6^/mL]).

### 2.3 Environmental exposure data

We used meteorological data provided by the Korea Meteorological Administration, South Korea’s national weather service, through the Weather Data Open Portal (https://data.kma.go.kr/resources/html/en/aowdp.html) for the entire study period. We extracted weather data for Seoul from the Weather Data Open Portal, where all analyses were performed. These data included daily measurements of temperature in degrees Celsius (°C) and relative humidity (%), based on 24-h averages. For each semen sample, the weather data were mapped to the date of collection. The variables included: (1) daily average temperature, (2) daily maximum temperature, (3) daily minimum temperature, (4) daily average relative humidity, and (5) daily minimum relative humidity. Only one missing value for the minimum temperature was addressed by averaging the values from the preceding and following days. Two biologically relevant time periods, 0–90 and 70–90 days before ejaculation, were defined, corresponding to key phases of spermatogenesis. For each patient, the average temperature and humidity during these windows were calculated and analyzed in relation to the semen quality.

### 2.4 Statistical analysis

In this study, data were organized based on two lag periods (70 and 90 days before ejaculation) to analyze the relationship between semen quality parameters (volume, concentration, morphology, motility, and TSC) and environmental variables (temperature and humidity). To address intra-subject correlations from repeated measures, we used generalized linear mixed-effects models to assess the effects of temperature and humidity on semen quality. Specifically, the Tweedie distribution with a log link function and variance power parameter of 1.5 was applied. This approach is appropriate for semi-continuous data with a point mass at zero and continuous distribution over positive values. In this modeling framework, the exponential of the regression coefficients represents the relative expected mean (REM), reflecting the multiplicative change in the overall expected value of the semicontinuous outcome with a one-unit increase in the covariate. Fixed effects in the model included temperature, humidity, season, and COVID-19 phase (before, during and after COVID-19). The overall study period was categorized into three phases based on national COVID-19 milestones: (1) before COVID-19: January 1, 2018–February 28, 2020; (2) during COVID-19: March 1, 2020–August 31, 2022; and (3) after COVID-19: September 1, 2022–December 31, 2023. This classification reflects major shifts in population behavior and environmental exposure that may have influenced the male reproductive health. We defined the pandemic period as beginning in March 2020, reflecting the onset of social restrictions, quarantine policies, and pandemic-related stress, and ending in September 2022, which marked the lifting of distancing restrictions and the return to daily routines. These cutoffs were based on infection statistics from the Seoul region in conjunction with the timing of major public health interventions, including the nationwide social distancing in South Korea, as supported by previous literature (18, 19). Environmental variables recorded as near-zero string values were converted to decimal values for the numerical analysis. Other missing values were excluded before the analysis. All analyses were conducted after adjusting for age to control for its potential effect on semen quality. All statistical tests were two-sided, and a significance level of 0.05 was used. Statistical analyses were conducted using Python 3.12.3 and R 4.4.2.

## 3 Results

### 3.1 Participant characteristics

A total of 2,672 semen analysis records from 1,287 unique individuals were included. Of these, 54.6% (n = 703) underwent multiple semen analyses, ranging from 1 to 18, with a median of two visits. Repeated measures from the same individuals were retained to assess within-subject variability and enable lagged environmental exposure modeling (**Table 1**). Semen volume was significantly higher in the before COVID-19 period compared to during and after COVID-19 periods (3.07 vs. 2.79 and 2.73 mL, respectively; p < 0.001).

**Table 1:** Descriptive characteristics of semen analysis records across different periods of the COVID-19 pandemic (N = 2,672).

	Total (N=2672)	Before COVID-19 (N=692)	During COVID-19 (N=1057)	After COVID-19 (N=923)	p-value
Age at time of test (years)	34.76 ± 8.16	34.02 ± 7.37	35.24 ± 8.08	34.78 ± 8.76	<0.01**
Volume (mL)	2.84 ± 1.63	3.07 ± 1.66	2.79 ± 1.59	2.73 ± 1.65	<0.001***
Sperm concentration (million/mL)	73.85 ± 75.14	57.43 ± 57.30	78.29 ± 80.10	81.9 ± 79.19	<0.001***
Normal morphology (%)	2.19 ± 1.09	2.33 ± 1.36	2.15 ± 1.01	2.14 ± 0.93	<0.001***
Motility (%)	44.78 ± 22.18	42.53 ± 21.65	46.39 ± 22.28	44.64 ± 22.34	<0.001***
Total Sperm Count (million/mL)	202.94 ± 217.84	178.40 ± 195.29	212.41 ± 233.09	210.50 ± 214.60	<0.01**

Data are expressed as mean ± standard deviation.

***p < 0.001, **p < 0.01, *p < 0.05.

**Figure 1:**
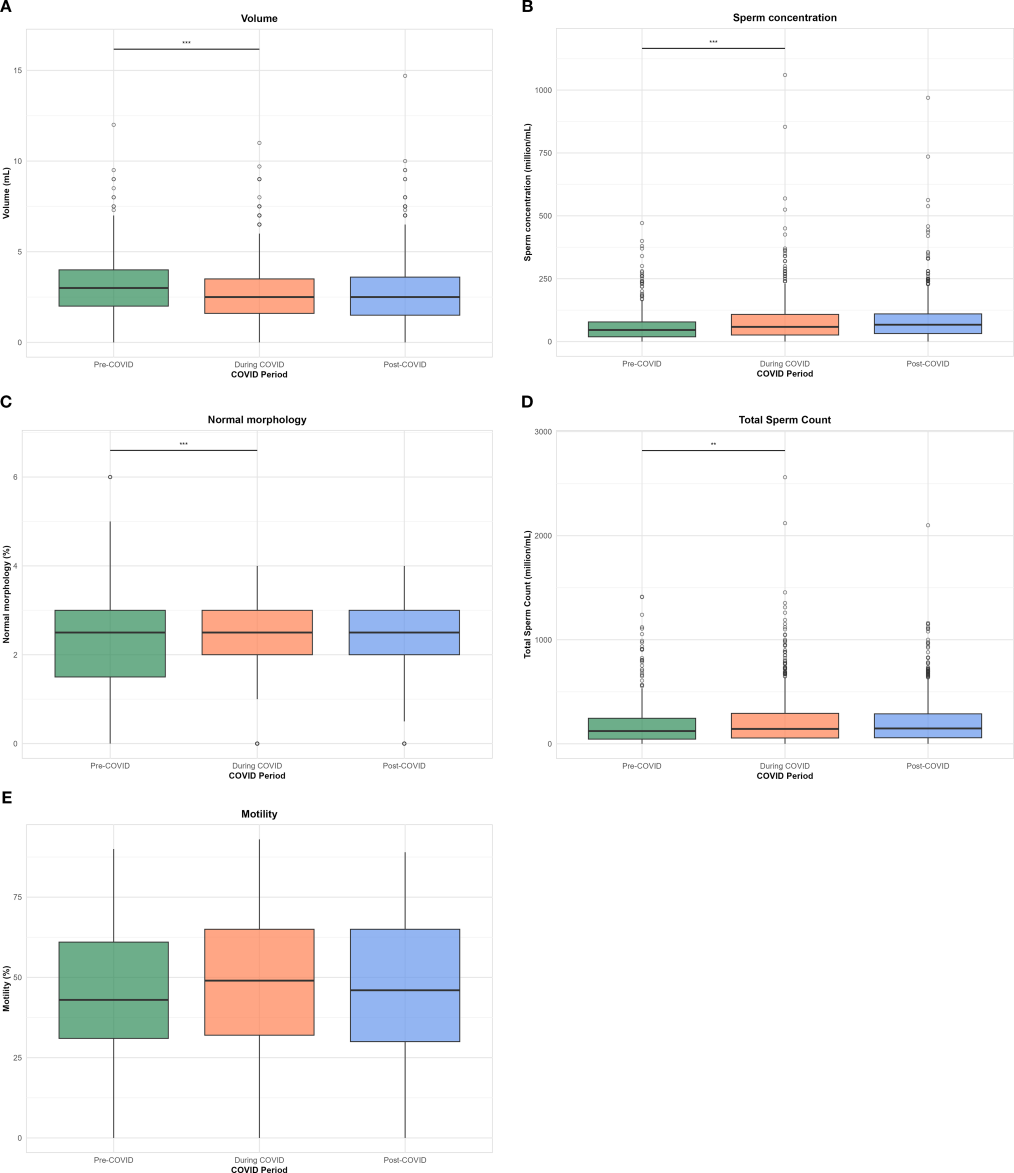
Boxplots of semen parameters across COVID-19 periods (pre-, during-, and post-COVID-19). Each panel represents the distribution of: **(A)** ejaculate volume (vol), **(B)** sperm count, **(C)** sperm morphology score (SM), **(D)** total sperm count (TSC), and **(E)** sperm motility (SM). Statistical significance was assessed using ANOVA with *post hoc* pairwise comparison; p < 0.05 (*), < 0.01 (**), < 0.001 (***), and < 0.0001 (****); ns, not significant.

Sperm concentration and total sperm count were also significantly elevated during and after the pandemic compared to the before COVID-19 period (p < 0.001 and p = 0.002, respectively). Motility and normal morphology rates showed statistically significant but modest differences across time periods (p < 0.001 for both), with slightly higher motility during the COVID-19 period. The distribution of semen quality parameters across the before, during and after COVID-19 periods is presented in **Figure 1**.

### 3.2 Association between climatic variables and semen quality parameters based on repeated-measures analysis

As the interaction effects between the COVID-19 phase and climatic variables were not statistically significant, we confirmed that temporal variations in climatic conditions were largely independent of the pandemic phase. Our findings suggest that elevated ambient temperature 70–90 days before semen collection was significantly associated with reduced sperm concentration, whereas no significant associations were observed for other semen parameters (**Table 2**). Specifically, higher average (relative expected mean (REM) = 0.966, 95% CI: 0.935–0.997, p = 0.033), maximum (REM = 0.965, 95% CI: 0.934–0.996, p = 0.029), and minimum (REM = 0.966, 95% CI: 0.937–0.997, p = 0.032) temperatures in the 70–90 day window were each significantly associated with reduced sperm concentration. No consistent or statistically significant associations were observed between semen parameters and humidity variables. These results suggest that early spermatogenesis is particularly susceptible to climatic stressors such as high temperatures.

**Table 2:** Relative expected mean for continuous semen parameters in association with lagged temperature and humidity exposures.

	Volume		Sperm concentration		Sperm morphology		Motility		Total Sperm Count	
	REM (95% CI)	p-value	REM (95% CI)	p-value	REM (95% CI)	p-value	REM (95% CI)	p-value	REM (95% CI)	p-value
AvgT (0-90d)	1.003 (0.981-1.025)	0.801	0.967 (0.933-1.002)	0.065	1.001 (0.963-1.041)	0.945	1.001 (0.971-1.033)	0.932	0.972 (0.934-1.012)	0.170
MaxT (0-90d)	1.002 (0.98-1.025)	0.841	0.966 (0.931-1.002)	0.064	1.001 (0.963-1.042)	0.942	1 (0.969-1.032)	0.997	0.971 (0.933-1.012)	0.163
MinT (0-90d)	1.003 (0.982-1.025)	0.764	0.967 (0.933-1.001)	0.059	1.002 (0.965-1.041)	0.925	1.002 (0.972-1.033)	0.884	0.973 (0.936-1.011)	0.165
AvgH (0-90d)	1.001 (0.974-1.029)	0.932	0.986 (0.942-1.032)	0.540	0.981 (0.936-1.028)	0.417	1.016 (0.979-1.055)	0.395	0.982 (0.934-1.033)	0.486
MinH (0-90d)	0.998 (0.976-1.021)	0.871	0.993 (0.957-1.031)	0.716	0.984 (0.947-1.022)	0.409	1.016 (0.985-1.047)	0.320	0.987 (0.947-1.029)	0.550
AvgT (70-90d)	1.002 (0.983-1.021)	0.871	0.966 (0.935-0.997)	<0.05*	0.98 (0.947-1.014)	0.253	0.986 (0.959-1.013)	0.302	0.974 (0.94-1.009)	0.143
MaxT (70-90d)	1 (0.98-1.019)	0.970	0.965 (0.934-0.996)	<0.05*	0.978 (0.945-1.013)	0.213	0.984 (0.957-1.012)	0.261	0.971 (0.937-1.006)	0.109
MinT (70-90d)	1.003 (0.984-1.022)	0.757	0.966 (0.937-0.997)	<0.05*	0.983 (0.95-1.017)	0.315	0.987 (0.961-1.014)	0.347	0.976 (0.942-1.01)	0.159
AvgH (70-90d)	1.008 (0.987-1.03)	0.466	0.987 (0.953-1.023)	0.483	1 (0.963-1.038)	0.991	1.001 (0.971-1.032)	0.935	0.989 (0.951-1.029)	0.583
MinH (70-90d)	1.003 (0.981-1.025)	0.801	0.966 (0.931-1.002)	0.064	1.001 (0.963-1.042)	0.942	1 (0.969-1.032)	0.997	0.971 (0.933-1.012)	0.163

REM, relative expected mean; all correlation coefficients are adjusted for age.

AvgT (0-90d): Average Temperature Day 0 to 90, AvgT (79-90d): Average Temperature Day 70 to 90.

MaxT (0-90d): Maximum Temperature Day 0 to 90, MaxT (70-90d): Maximum Temperature Day 70 to 90.

MinT (0-90d): Minimum Temperature Day 0 to 90, MinT (70-90d): Minimum Temperature Day 70 to 90.

AvgH (0-90d): Average Humidity Day 0 to 90, AvgH (79-90d): Average Humidity Day 70 to 90.

MaxH (0-90d): Maximum Humidity Day 0 to 90, MaxH (70-90d): Maximum Humidity Day 70 to 90.

MaxH (0-90d): Minimum Humidity Day 0 to 90, MinH (70-90d): Minimum Humidity Day 70 to 90.

Volume (mL); Sperm concentration (million/mL); Morphology (% normal forms); Motility (%); Total Sperm Count (million/mL).

***p < 0.001, **p < 0.01, *p < 0.05.

### 3.3 Association between climatic variables and semen quality parameters across different periods of the COVID-19 pandemic

During the COVID-19 period, a consistent and statistically significant inverse relationship was observed between the multiple temperature indicators and TSC. Average, maximum, and minimum temperature (0–90 days prior) were significantly and negatively associated with sperm concentration and TSC (p < 0.001), with REMs for sperm concentration ranging from 0.863 to 0.890 (**Table 3**). Average humidity also showed a negative association with sperm concentration (REM =: 0.868; p < 0.001). No significant associations were found for morphology or motility. These associations remained robust in the 70–90 d pre-ejaculation window. In contrast, during the before COVID-19 and after COVID-19 periods, none of the temperature or humidity variables showed statistically significant associations with any of the semen parameters.

**Table 3:** Association between climatic variables and semen quality parameters across different periods of the COVID-19 pandemic.

	Volume		Sperm concentration		Sperm morphology		Motility		Total Sperm Count	
	REM (95% CI)	p-value	REM (95% CI)	p-value	REM (95% CI)	p-value	REM (95% CI)	p-value	REM (95% CI)	p-value
Before COVID-19										
AvgT (0-90d)	1.044 (0.998-1.092)	0.061	1.025 (0.952-1.104)	0.505	1.004 (0.924-1.092)	0.918	0.977 (0.916-1.041)	0.469	1.058 (0.974-1.149)	0.184
MaxT (0-90d)	1.044 (0.998-1.092)	0.059	1.024 (0.952-1.103)	0.523	1.005 (0.924-1.092)	0.911	0.976 (0.916-1.04)	0.454	1.057 (0.973-1.148)	0.189
MinT (0-90d)	1.043 (0.998-1.091)	0.064	1.025 (0.952-1.103)	0.519	1.005 (0.925-1.093)	0.899	0.978 (0.917-1.042)	0.486	1.056 (0.973-1.147)	0.194
AvgH (0-90d)	1.062 (0.987-1.142)	0.107	1.043 (0.927-1.173)	0.484	1.016 (0.885-1.166)	0.820	1.005 (0.906-1.115)	0.927	1.087 (0.953-1.241)	0.213
MinH (0-90d)	1.049 (0.979-1.125)	0.176	1.043 (0.931-1.167)	0.468	1.001 (0.877-1.142)	0.990	0.995 (0.9-1.099)	0.919	1.075 (0.948-1.22)	0.261
AvgT (70-90d)	1.018 (0.978-1.059)	0.392	1.039 (0.973-1.109)	0.250	0.948 (0.88-1.021)	0.155	0.971 (0.917-1.028)	0.315	1.059 (0.984-1.139)	0.124
MaxT (70-90d)	1.016 (0.976-1.057)	0.441	1.043 (0.977-1.113)	0.207	0.943 (0.876-1.015)	0.119	0.970 (0.916-1.027)	0.297	1.060 (0.986-1.14)	0.112
MinT (70-90d)	1.019 (0.979-1.06)	0.356	1.034 (0.969-1.103)	0.312	0.953 (0.885-1.027)	0.206	0.972 (0.918-1.03)	0.339	1.055 (0.982-1.134)	0.145
AvgH (70-90d)	1.050 (0.994-1.109)	0.080	0.968 (0.886-1.058)	0.478	1.029 (0.926-1.144)	0.594	0.987 (0.91-1.07)	0.747	1.016 (0.92-1.122)	0.756
MinH (70-90d)	1.044 (0.99-1.102)	0.112	0.967 (0.887-1.054)	0.447	1.034 (0.931-1.149)	0.532	0.981 (0.906-1.063)	0.645	1.010 (0.917-1.113)	0.836
During COVID-19										
AvgT (0-90d)	0.962 (0.928-0.996)	<0.05*	0.887 (0.837-0.94)	<0.001***	1.014 (0.949-1.084)	0.677	0.999 (0.95-1.05)	0.957	0.867 (0.812-0.926)	<0.001***
MaxT (0-90d)	0.959 (0.925-0.994)	<0.05*	0.886 (0.835-0.94)	<0.001***	1.013 (0.947-1.084)	0.703	0.997 (0.947-1.049)	0.901	0.863 (0.807-0.924)	<0.001***
MinT (0-90d)	0.964 (0.932-0.998)	<0.05*	0.890 (0.841-0.941)	<0.001***	1.015 (0.952-1.082)	0.648	1 (0.952-1.05)	0.991	0.871 (0.818-0.929)	<0.001***
AvgH (0-90d)	0.990 (0.949-1.032)	0.635	0.868 (0.811-0.93)	<0.001***	1.011 (0.936-1.092)	0.777	1.002 (0.944-1.062)	0.956	0.871 (0.806-0.942)	<0.001***
MinH (0-90d)	0.994 (0.96-1.028)	0.719	0.892 (0.842-0.944)	<0.001***	1.013 (0.951-1.079)	0.687	1.003 (0.955-1.052)	0.917	0.898 (0.842-0.957)	<0.01**
AvgT (70-90d)	0.974 (0.944-1.005)	0.098	0.890 (0.844-0.938)	<0.001***	1.003 (0.945-1.066)	0.912	0.993 (0.949-1.04)	0.779	0.876 (0.825-0.929)	<0.001***
MaxT (70-90d)	0.971 (0.941-1.002)	0.071	0.885 (0.839-0.933)	<0.001***	1.002 (0.943-1.065)	0.945	0.993 (0.948-1.04)	0.757	0.869 (0.819-0.923)	<0.001***
MinT (70-90d)	0.976 (0.947-1.006)	0.122	0.895 (0.849-0.942)	<0.001***	1.005 (0.948-1.066)	0.862	0.994 (0.951-1.039)	0.794	0.882 (0.832-0.935)	<0.001***
AvgH (70-90d)	1.002 (0.969-1.035)	0.923	0.939 (0.887-0.993)	<0.05*	1.024 (0.962-1.09)	0.462	0.994 (0.948-1.042)	0.793	0.942 (0.884-1.004)	0.064
MinH (70-90d)	1.005 (0.978-1.033)	0.708	0.957 (0.913-1.004)	0.071	1.019 (0.967-1.073)	0.482	0.996 (0.958-1.036)	0.851	0.964 (0.914-1.016)	0.171
After COVID-19										
AvgT (0-90d)	1.005 (0.969-1.042)	0.797	1.007 (0.949-1.069)	0.819	0.994 (0.94-1.051)	0.839	1.014 (0.965-1.067)	0.580	1.021 (0.955-1.091)	0.547
MaxT (0-90d)	1.003 (0.966-1.042)	0.859	1.007 (0.947-1.07)	0.831	0.994 (0.939-1.053)	0.848	1.015 (0.963-1.069)	0.583	1.019 (0.951-1.091)	0.599
MinT (0-90d)	1.006 (0.971-1.042)	0.733	1.007 (0.951-1.067)	0.809	0.994 (0.941-1.049)	0.825	1.014 (0.966-1.065)	0.580	1.022 (0.959-1.09)	0.500
AvgH (0-90d)	1.029 (0.978-1.083)	0.264	1.014 (0.931-1.104)	0.752	0.983 (0.907-1.066)	0.681	0.998 (0.928-1.074)	0.958	1.067 (0.972-1.171)	0.174
MinH (0-90d)	1.022 (0.982-1.062)	0.286	1.016 (0.952-1.085)	0.629	0.992 (0.932-1.055)	0.789	1.008 (0.952-1.066)	0.793	1.053 (0.98-1.131)	0.159
AvgT (70-90d)	1.004 (0.973-1.036)	0.803	1.013 (0.961-1.067)	0.639	0.991 (0.944-1.042)	0.734	0.986 (0.943-1.03)	0.528	1.032 (0.974-1.093)	0.286
MaxT (70-90d)	1.002 (0.97-1.035)	0.907	1.014 (0.961-1.07)	0.606	0.992 (0.943-1.044)	0.758	0.984 (0.941-1.03)	0.494	1.030 (0.971-1.093)	0.320
MinT (70-90d)	1.006 (0.975-1.037)	0.723	1.012 (0.962-1.065)	0.650	0.991 (0.945-1.04)	0.722	0.988 (0.946-1.031)	0.573	1.033 (0.977-1.093)	0.257
AvgH (70-90d)	1.013 (0.975-1.053)	0.517	1.006 (0.943-1.074)	0.846	1.001 (0.941-1.064)	0.987	0.992 (0.938-1.05)	0.790	1.038 (0.966-1.115)	0.310
MinH (70-90d)	1.014 (0.984-1.045)	0.366	1.008 (0.957-1.06)	0.772	0.998 (0.952-1.047)	0.938	1.003 (0.96-1.047)	0.901	1.038 (0.982-1.098)	0.190

REM, relative expected mean; all correlation coefficients are adjusted for age.

***p < 0.001, **p < 0.01, *p < 0.05.

### 3.4 Relative differences in semen quality parameters across different periods of the COVID-19 pandemic

Compared to the before COVID-19 period, the during COVID-19 period showed significantly lower semen volume (REM = 1.114, 95% CI: 1.063–1.167, p < 0.001), higher sperm concentration (REM = 0.744, 95% CI: 0.691–0.800, p < 0.001), and higher TSC (REM 0.867, 95% CI: 0.787–0.954, p = 0.034) (**Table 4**). Similarly, compared to before COVID-19, the after COVID-19 period was also associated with lower semen volume (REM = 1.135, 95% CI: 1.080–1.193, p < 0.001) and higher sperm concentration (REM = 0.755, 95% CI: 0.696–0.818, p < 0.001), whereas TSC showed no statistically significant difference. No significant differences were observed between the during COVID-19 and after COVID-19 periods for any of the semen quality parameters.

**Table 4:** Relative differences in semen quality parameters across different periods of the COVID-19 pandemic.

	Volume		Sperm concentration		Sperm morphology		Motility		Total Sperm Count	
	REM (95% CI)	p-value	REM (95% CI)	p-value	REM (95% CI)	p-value	REM (95% CI)	p-value	REM (95% CI)	p-value
During vs. after COVID-19	1.019 (0.963-1.078)	1.000	1.015 (0.929-1.109)	1.000	1.006 (0.927-1.092)	1.000	1.037 (0.97-1.109)	0.810	1.058 (0.952-1.176)	0.798
Before vs. during COVID-19	1.114 (1.043-1.191)	<0.01**	0.744 (0.691-0.8)	<0.001***	1.087 (0.988-1.196)	0.191	0.922 (0.864-0.984)	0.070	0.867 (0.787-0.954)	<0.05*
Before vs. after COVID-19	1.135 (1.054-1.224)	<0.001***	0.755 (0.696-0.818)	<0.001***	1.093 (0.99-1.207)	0.163	0.956 (0.892-1.025)	0.669	0.917 (0.821-1.025)	0.482

REM, relative expected mean; all correlation coefficients are adjusted for age.

***p < 0.001, **p < 0.01, *p < 0.05.

## 4 Discussion

In this retrospective cohort study, we evaluated the associations between ambient temperature, humidity, and semen quality across different time windows using national meteorological data and 2,671 clinical semen analysis records from 1,287 men. We further stratified the analysis by before, during, and after COVID-19 periods to capture environmental and societal interactions that may have affected male reproductive health.

Our findings confirm previous studies showing that elevated ambient temperature adversely affects semen quality, particularly sperm concentration and TSC, with significant associations observed for average temperature during both the 0–90 d and 70–90 d windows prior to sperm analysis (20). Notably, this negative association was most pronounced during the COVID-19 period. These findings support the hypothesis that the early stages of spermatogenesis are particularly vulnerable to thermal stress (21–23). These patterns align with existing evidence that high ambient temperatures can impair spermatogenesis, potentially through mechanisms involving oxidative stress, impaired thermoregulation, and hormonal disruption (24). Collectively, our findings highlight the complex relationship between climatic conditions and male reproductive functions.

Several studies, including a systematic review and meta-analysis by Ashonibare et al. and a longitudinal analysis by Holtmann et al., have suggested the negative effects of severe acute respiratory syndrome coronavirus 2 infection on semen quality (25–27). These studies emphasized the biological mechanisms such as direct viral invasion, oxidative stress, systemic inflammation, and reduced testosterone levels. Our study did not directly assess the impact of confirmed severe acute respiratory syndrome coronavirus 2 infection; however, we examined population-level trends in semen parameters across the before, during, and after COVID-19 periods, which potentially reflect changes in behavior, environmental exposure, or data characteristics. Semen parameters, including sperm concentration, and TSC, significantly improved during the COVID-19 period, whereas semen volume decreased, and sperm motility remained relatively stable. Notably, this was the only time window in which these parameters showed statistically significant negative associations with both ambient temperature and humidity.

One possible explanation for this discrepancy is the shift in patients’ lifestyles and environmental exposure during the pandemic. Reduced occupational stress, decreased air pollution, and increased rest because of social distancing policies in Korea may have exerted beneficial effects on male reproductive health. Furthermore, the male reproductive system may become more sensitive to environmental stressors, such as elevated temperature and humidity. Prior studies have reported transient improvements in select semen parameters during the COVID-19 pandemic; however, these were generally limited to small-scale surveys or secondary findings within broader analyses that primarily emphasized the negative impact of the pandemic (28, 29). To our knowledge, this is the first study to systematically document enhanced sperm concentration and TSC during and after the COVID-19 pandemic, while also showing that ambient temperature and humidity had the strongest negative associations with semen quality during the COVID-19 period. These findings highlight a unique temporal window in which reproductive health was shaped by both societal changes and environmental stressors, offering novel insights into the interaction between climate dynamics and male fertility.

However, contrary to earlier studies reporting strong negative associations between humidity and semen parameters (7, 30), our findings did not reveal consistent relationships between humidity indicators and semen quality across all models. While some models showed associations between average/minimum humidity and sperm motility, these findings were inconsistent and lost statistical significance after adjusting for repeated measures. This reinforces the interpretation that ambient temperature, rather than humidity, is the more robust climatic determinant of spermatogenesis.

### 4.1 Strengths and limitations

A major strength of our study is the large sample size and the inclusion of repeated semen analyses in over half of the participants, enabling within-subject comparisons and lagged exposure modeling. Furthermore, all semen analyses were conducted at a single tertiary center using standardized protocols and rigorous quality control measures, thereby minimizing inter-laboratory variability and enhancing internal consistency. Another notable strength is the use of nationally standardized meteorological data across a 6-year period (2018–2024), which captured substantial seasonal and year-to-year variations in temperature and humidity. This created a robust natural experimental framework for assessing the real-world environmental impacts on semen quality, particularly during extreme weather events such as heatwaves and cold spells. Stratification by pandemic phase further enabled the study to account for not only environmental exposures but also societal, behavioral, and policy-related influences that may confound or mediate the reproductive outcomes. This multidimensional approach enhances the interpretability and generalizability of our findings in clinical and public health contexts.

Nevertheless, some limitations of this study need to be discussed. First, the study lacked individual-level data on COVID-19 infection status, viral strain, vaccination history and comorbidities, which could influence male reproductive function. Future studies should incorporate these variables to allow adjustment for these potential confounders. In addition, detailed information on personal environmental exposures, such as indoor climate control, occupational heat exposure, smoking habits, and job-related stressors, was unavailable. Meteorological exposures were assigned based on the hospital’s geographic location, assuming that all participants were exposed to the same regional weather conditions, which may not accurately reflect the participants’ actual residential or occupational environments. Finally, as the cohort primarily consisted of subfertile men and patients with cancer undergoing fertility preservation, the generalizability of our findings to the broader male population may be limited.

Further studies incorporating more granular geographic data, including individual-level temperature and humidity tracking, are required to accurately estimate true environmental exposures. The inclusion of occupational and indoor climate data may also help refine exposure-response relationships. Given the global trend of rising temperatures, future research should investigate the long-term reproductive health implications of climate change in larger and more diverse populations. Furthermore, collecting comprehensive information on COVID-19 infection status, vaccination history, and relevant lifestyle factors, such as smoking, nutrition, stress, and physical activity, will be essential to distinguish direct biological effects from pandemic-related behavioral shifts.

## 5 Conclusion

In conclusion, overall semen quality improved during the COVID-19 pandemic; however, our findings indicate that this period was uniquely characterized by increased climatic sensitivity of spermatogenesis. This may reflect changes in environmental exposures and lifestyle patterns, underscoring the complex interplay between public health crises, human behavior, and male reproductive health. Future research should incorporate more detailed indoor climate data and occupational exposures to fully elucidate these dynamics.

## Data Availability

The clinical datasets analyzed in this study are not publicly available due to institutional and ethical restrictions. Aggregated meteorological data used in the analysis are publicly available from the Korea Meteorological Administration (https://data.kma.go.kr). Further inquiries can be directed to the corresponding authors. Requests to access the datasets should be directed to Heeyon Kim, kimhy@yuhs.ac.
